# A Facile and Low-Cost Method to Enhance the Internal Quantum Yield and External Light-Extraction Efficiency for Flexible Light-Emitting Carbon-Dot Films

**DOI:** 10.1038/srep19991

**Published:** 2016-01-29

**Authors:** Z. C. Jiang, T. N. Lin, H. T. Lin, M. J. Talite, T. T. Tzeng, C. L. Hsu, K. P. Chiu, C. A. J. Lin, J. L. Shen, C. T. Yuan

**Affiliations:** 1 Department of Physics, Chung Yuan Christian University, Chung Li, 320, Taiwan; 2 Department of Biomedical Engineering, Chung Yuan Christian University, Chung Li, 320, Taiwan

## Abstract

Solution-processed, non-toxic carbon dots (CDs) have attracted much attention due to their unique photoluminescence (PL) properties. They are promising emissive layers for flexible light-emitting devices. To this end, the CDs in pristine aqueous solutions need to be transferred to form solid-state thin films without sacrificing their original PL characteristics. Unfortunately, solid-state PL quenching induced by extra non-radiative (NR) energy transfer among CDs would significantly hinder their practical applications in optoelectronics. Here, a facile, low-cost and effective method has been utilized to fabricate high-performance CD/polymer light-emitting flexible films with submicron-structured patterns. The patterned polymers can serve as a solid matrix to disperse and passivate CDs, thus achieving high internal quantum yields of 61%. In addition, they can act as an out-coupler to mitigate the waveguide-mode losses, approximately doubling the external light-extraction efficiency. Such CD/polymer composites also exhibit good photo-stability, and thus can be used as eco-friendly, low-cost phosphors for solid-state lighting.

Solution-processed colloidal nano-materials, such as CdSe quantum dots (QDs), graphene QDs and CDs, have attracted much attention due to their unique PL properties and can be used as emissive layers or wavelength-conversion phosphors for promising applications in flexible light-emitting devices^1^. Among them, cadmium-containing QDs are the most mature materials systems, in terms of materials synthesis, device fabrication, and understanding the photo-physical properties^2^. However, cadmium-containing QDs suffer from serious problems regarding the involvement of toxic components and the use of complex and expensive synthesis methods. Recently, a new class of eco-friendly colloidal nano-materials based on abundant, low-cost carbon elements, namely CDs and graphene QDs, has been discovered. This new class inherited some fascinating photo-physical and photo-chemical properties from their parental carbon compounds while exhibiting some unique and novel optoelectronic properties due to quantum confinement^3^. More importantly, the CDs can be prepared using low-cost raw materials as the carbon sources, such as waste foods, leaf and milk using a facile hydrothermal treatment^4^.

In addition to the benefits inherited from carbon-based nano-materials, such as the low-cost and abundant raw materials, simple synthesis and solution-processed manufacturing properties, the CDs also exhibit some unique photo-physical properties, including tunable PL properties spanning the whole visible spectrum^5^ and excellent photo-stability when dispersed in an aqueous solution.^6^ Thus, the CDs are expected to be employed as promising active layers for practical applications in the development of flexible light-emitting diodes^7^,^8^. However, most of the effort has focused on the fabrication methods and the characterization of the PL properties of CDs in their original aqueous solution, leaving the photo-physical properties of their solid-state forms unexplored. In general, luminescent colloidal QDs exhibit better PL emission behavior when dispersed in their original solution environment compared with that of colloidal QDs in solids^2^. In particular, for CDs without extra passivating ligands or outer shells, weak or even disappeared PL emission along with poor photo-stability are expected in the solid states at ambient environment, significantly hindering the potential uses in optoelectronic applications^9^.

After solvent evaporation, colloidal nano-materials tend to aggregate, thus introducing extra NR relaxation pathways, for example, energy transfer among nano-material ensembles, which is referred to as concentration-induced PL self-quenching or aggregation-induced PL self-quenching^10^. Previous reports have also demonstrated that the environment around the CDs can cause emission quenching, namely environment-induced quenching^4^,^11^. In addition to PL quenching, another detrimental effect induced by solid-state CDs is their poor photo-stability when exposed to the ambient environment. A general way to address this problem is to introduce a transparent solid dispersion, which can serve as a host matrix to spread the CDs while protecting them from the ambient environment^12^. Unfortunately, the introduction of a solid matrix would unavoidably cause another negative effect on emission behavior, namely, poor light-extraction efficiency due to the excitation of the waveguide-modes, leading to light-trapping within the relatively high-index host matrix, leading to the waveguide-mode losses^13^. As a result, it is essential to develop high-performance solid-state light-emitting thin films that can retain their original PL emission properties while mitigating the waveguide-mode losses, so they can serve as efficient phosphors for prospective applications in flexible light-emitting devices. The most commonly used phosphors for white light-emitting diodes are based on rare-earth doped phosphors or toxic cadmium-containing QDs. Therefore, it is necessary to develop eco-friendly phosphors based on the abundant elements that can be manufactured using facile and low-cost solution-processed methods.

This work prepared flexible light-emitting thin films, consisting of CDs and Poly(vinyl alcohol) (PVA) polymers, which can exhibit enhanced PL properties to further extend the utility of CDs in solution, in particular, to serve as down-conversion phosphors for flexible light-emitting devices. Such PVA polymer matrices can disperse and passivate CDs, thus playing a positive role in protecting CDs from concentration-induced PL self-quenching. However, the matrices unavoidably introduce a detrimental effect, waveguide-mode losses, owing to its planar geometry with a relatively high refractive index compared with that of the ambient environment. To address this problem, periodic micro-structures were patterned onto the surface of the PVA matrices, which can out-couple the waveguide-mode light to far-field emission. As a result, such CD/PVA composites with micro-structured patterns could be used as eco-friendly, low-cost and effective light-emitting phosphors.

## Results

**Figure 1:**
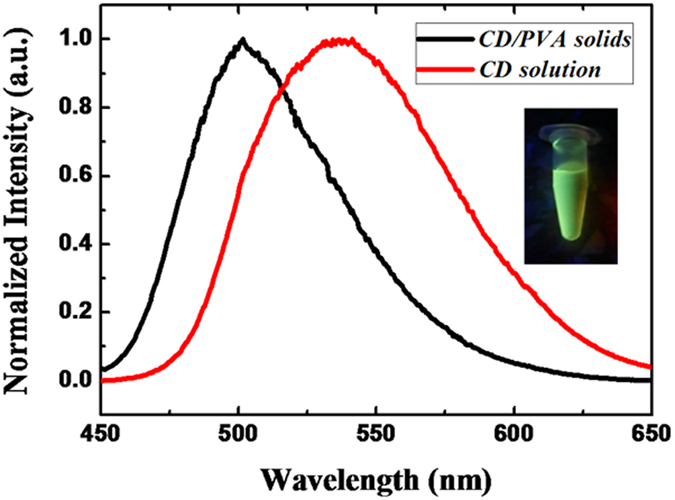
PL spectra for CDs dispersed in an aqueous solution and embedded into PVA matrices along with a photograph showing luminescent CDs in solution under UV irradiation.

Figure 1 shows the PL emission spectra with an excitation wavelength of ~405 nm for CDs dispersed in their original aqueous solution and embedded into PVA solid matrices. The inset shows a CD solution under UV irradiation. The quantum yields (QYs) of the PL emission for CDs in aqueous solution are ~0.17, which are obtained by an absolute-QY measurement based on an integrating sphere combined with a spectrometer and a photo-detector. For practical applications in light-emitting devices, the colloidal CDs need to be transferred into the solid-state phase for further utilization in active emissive layers or wavelength down-conversion phosphors for generating electroluminescence or photoluminescence^14^. To this end, the PL emission must be retained or even can be further enhanced for solid-state CDs. The commonly used phosphors for white light-emitting diodes contain rare-earth elements or colloidal QDs; QDs are toxic due to the presence of heavy-metal cadmium elements, and need to be replaced with inexpensive, abundant and non-toxic nano-materials^15^,^16^,^17^,^18^. To protect CDs from extra NR relaxation in the solid state, a rigid host matrix needs to be introduced. Figure 1 also shows the PL spectrum for solid-state CD/PVA composites. Interestingly, the PL spectrum of CD/PVA composites is shifted to the high-energy side, which is not typical for colloidal nano-materials upon transferring into solid states^19^. In general, solid-state PL self-quenching occurs due to the introduction of extra NR energy transfer from higher energy states to lower energy states among aggregated nano-materials ensembles, leading to the red-shift of the PL spectrum, significant PL-QY reduction and the shortening of the PL lifetime. This finding implies that no self-quenching interaction was introduced after the formation of the solid CD/PVA composites.

**Figure 2:**
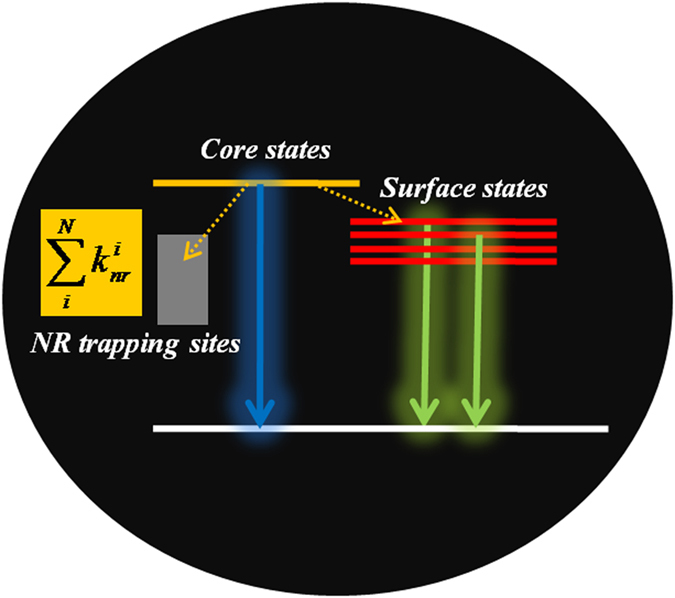
Schematic illustration of PL emission from CDs with multiple non-radiative decay rates.

The exact PL mechanism for CDs in aqueous solution is still under debate and might be related to both contribution of core electronic states and surface electronic states, as schematically illustrated in Fig. 2^20–23^. Yu *et al.* has proposed that the PL emission of CDs can be attributed to the core-state emission and the surface-state emission^24^. Here, we tentatively assigned this PL blue-shift to the passivation of the CDs by the PVA polymer matrices, which will be confirmed later using time-resolved PL spectroscopy. Once the NR defect sites can be mitigated, the ratio of PL emission between intrinsic core states (higher emission energy) and surface states (lower emission energy) might increase, leading to the blue-shift of the PL emission. A previous report has proposed that the surface hydroxyl group of PVA polymers might play an important role in interacting with the oxygen-containing functional groups around the CD surfaces to form rigid hydrogen bonds, leading to the suppression of NR recombination^25^.

**Figure 3:**
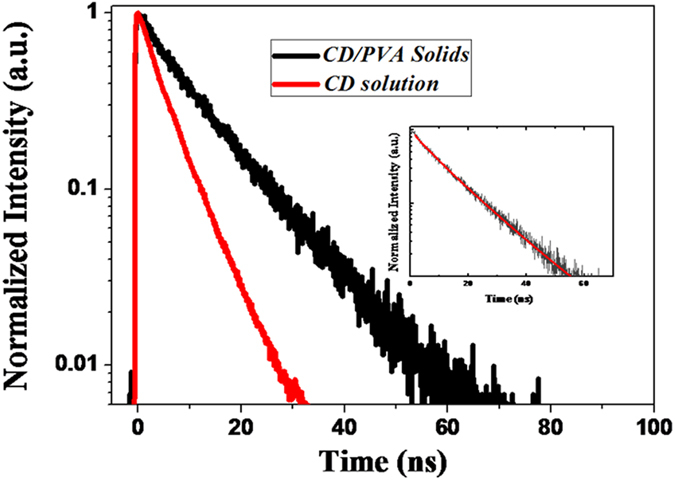
Time-resolved PL decay curves for liquid CDs and solid CD/PVA composites together with the fitting results for solid composites in the inset.

To prove the aforementioned hypothesis, time-resolved PL measurements were performed for CDs in an aqueous solution and in the solid state, as shown in Fig 3. The PL lifetime can be expressed by 
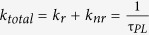
, where 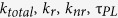 represent the total decay rats, the radiative decay rate, the NR decay rate, and the PL lifetime, respectively. When the NR decay rate can be suppressed due to the surface passivation of the NR defect sites, the total decay rate decreases, leading to the PL lifetime lengthening. Indeed, the PL lifetime of CD/PVA composites can be significantly lengthened compared with that of the CDs in an aqueous solution. To gain further insight into the PL decay dynamics of CDs, the PL decay curves were fitted using a stretched exponential function, 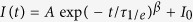, where 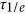
denotes the PL decay time (the time after the PL intensity drops to 1/e of its initial intensity, 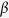 is a dispersion factor and 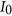
is the background noise. The corresponding average lifetime can be determined by 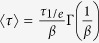, where Γ is the gamma function. The inset shown in Fig. 3 is the PL decay profile with a stretched exponential fitting for solid CD/PVA films. In general, the stretched-exponential PL decay curves can be observed for the light emitters with the dispersed NR decay rates, which can be expressed by 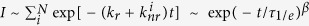
26,27. As a result, after the passivation of the surface traps, the PL decay lifetime can be lengthened along with the increase of the dispersion factor due to the reduction of the NR rates and their rate distribution^26^,^27^. The fitting parameters obtained from the stretched exponential functions are 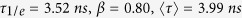 for CDs in an aqueous solution and 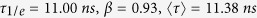 for solid CD/PVA composites. Such an experimental observation can provide more evidence for the surface passivation of CDs embedded into the PVA polymer matrices.

To compare the difference in the QYs for CDs in an aqueous solution and in the solid state, we used a simple equation to estimate the QYs of solid CD/PVA composites, 
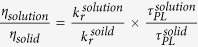
. The radiative decay rate of a spontaneous emission depends on the refractive index of the medium around the light emitters, which is known as the dielectric effect, 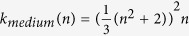 ; thus, we expect 
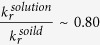
28. The variation of the PL lifetime can be directly obtained by our time-resolved PL experiments, which is 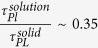. As a result, the QYs of the CD/PVA composites can be determined to be ~0.61 (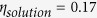, determined by an integrating sphere), which is ~3.6-fold enhancement as compared with CDs in an aqueous solution.

**Figure 4:**
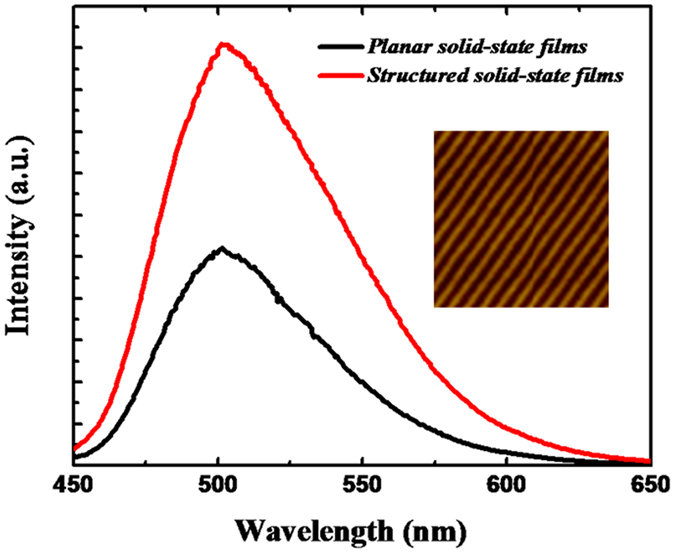
PL spectra for solid CD/polymer composites with and without the patterns of the grating structures along with an AFM imaging of the grating patterns in the inset.

Despite the aforementioned benefits arising from polymer matrices, solid-state composites would suffer from other issues related to the poor light-extraction efficiency due to relatively high refractive index compared with that of the air environment and their planar geometry. In a planar configuration, the PL emission would be confined within the planar waveguides due to total internal reflection, resulting in waveguide-mode losses^29^. To mitigate such waveguide-mode losses, Bragg gratings or some other wavelength-scale photonic structures have been utilized to extract the waveguide-mode light^29^,^30^,^31^,^32^,^33^. Unfortunately, the fabrication of well-defined grating structures necessitates expensive equipment or involves complex techniques, such as electron-beam lithography, which would significantly increase the fabrication cost. To overcome these shortcomings, we directly patterned the submicron-grating structure on the surface of the CD/PVA composites by replicating the patterns from a common digital versatile disc. The submicron-patterns with a periodicity of ~700 nm are revealed by the AFM imaging, as shown in the insert of Fig. 4. The trapped light within the waveguide modes can be out-coupled into free space by introducing periodic grating structures, which can compensate for the momentum mismatch between the waveguide-mode light and far-field radiation. This behavior can be described using the Bragg scattering equation, 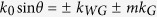, where 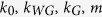 represent the wave-vector for free-space light, the waveguide-mode light, the periodic grating, and the order of scattering, respectively. As shown in Fig. 4, the PL emission can be enhanced by a factor of ~1.96 upon patterning the grating structures.

**Figure 5:**
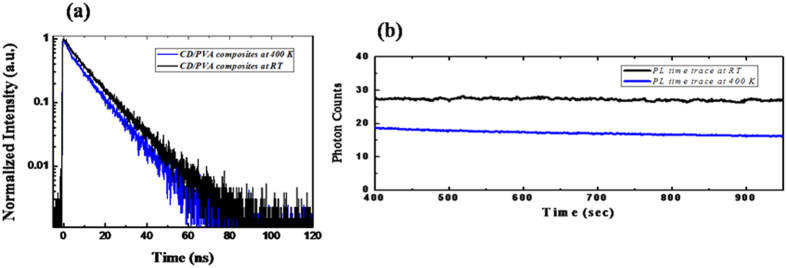
(**a**) Time-resolved PL decay profiles for solid CD/PVA composites at room temperature and 400 K and (**b**) the PL intensity as a function of observation time for solid composites at room temperature and 400 K.

The working temperature for phosphors for white light-emitting diodes is much higher than room temperature. As a result, thermal stability is another important materials property for light-emitting phosphors. We also investigated the PL properties and thermal stability at an elevated temperature. Figure 5a shows the PL decay profile of the CD/PVA composites at room temperature and high temperature (~400 K). In general, the PL lifetimes significantly decreased upon the introduction of extra NR decay pathways at the elevated temperature. The PL lifetime for solid CD/PVA composites was reduced slightly, indicating only a small increase in the NR recombination rates upon raising the temperature to ~400 K. As shown in Fig. 5b, the PL emission is stable at room temperature and the PL intensity slightly decreased along with a small degradation with time at the elevated temperature, implying their better thermal stability and photo-stability for those solid composites. A previous report also demonstrated that the surface NR defects play an important role in the photo-stability of colloidal CdTe QDs^34^. After the surface passivation of the QD surfaces, photo-stability was improved.

## Discussion (Conclusion)

In summary, high-performance solid-state luminescent thin films were fabricated by a facile and low-cost method based on non-toxic and abundant CDs embedded into PVA polymer matrices with sub-micron patterns. With the steady-state and time-resolved PL spectroscopic measurements, we found that, upon incorporating with polymer matrices, the PL emission spectrum of solid CD/PVA composites is blue shifted, which is accompanied by PL lifetime lengthening and near single-exponential decay profile, indicating the passivation of the surface NR defect states. In addition, a simple imprinting method was used to transfer the grating patterns to the PVA surface, thus enhancing the light-extraction efficiency. As a result, such patterned CD/PVA composites can not only enhance the internal quantum yields but also mitigate the waveguide-mode losses and exhibit stable PL emission. These composites are promising materials for use as wavelength-conversion phosphors in flexible light-emitting devices.

## Methods

### Preparation of CD/PVA composites with grating patterns

The precursor solution for CD synthesis was 1 g citric acid and 2 g urea dissolved in 20 ml of de-ionized water. The precursor solution was heated by a 700 W domestic microwave oven for 3.5 minutes. The solution changed from transparent to dark-brown clustered solids. Those solids were dissolved in 40 ml of de-ionized water and centrifuged to remove the large agglomerated particles at 6200 rpm for 20 minutes. Finally, the as-prepared solution was filtrated by syringe filter (0.22 μm). For the preparation of the PVA solution, 0.798 g of PVA (Mw=30,000–70,000 g) was added to 18 ml of de-ionized water. The resulting mixture was heated at 95 °C and stirred at 65 rpm for 30 minutes until the polymers were completely dissolved in the de-ionized water. The transparent solution was then cooled to room temperature. To prepare the solid films of the CD/PVA composites, both CDs and PVA solution were mixed, then poured into an aluminum dish and dried at 50 °C for 12 hours under vacuum. The solidified CD/PVA thin films were detached from the aluminum dish. To imprint the grating patterns onto the surface of the CD/PVA composites, the mixture solution of the CDs and the PVA polymer were deposited onto a commercial digital versatile disc, which served as a mold for the grating patterning. After polymer solidification, the grating patterns were transferred to the PVA polymers, which are confirmed by atomic force microscopy (Park System, XE-100).

### Spectroscopic characterization

Time-integrated and time-resolved PL spectroscopy was performed using a home-made confocal microscope. The excitation light provided by a pulsed diode laser emission at ~405 nm was focused onto the samples with a microscope objective. A suitable long-pass edge filter was used to block the excitation laser while guiding the PL emission to a spectrometer. The PL emission was collected by either a CCD camera for the PL spectrum or by a PMT (IRF~200 ps) for the time-resolved PL measurement. The time-correlated single-photon counting curves for time-resolved PL spectroscopy were obtained using a time correlator (HydroHarp 400, PicoQuant, Germany). The quantum yields of the CDs in aqueous solution was determined based on the absolute-QY measurement, which combines an integrating sphere, a spectrometer and a photo-detector (Horiba). For this purpose, two samples were prepared, which involve CDs in an aqueous solution as experimental samples and pure solvent in blank cuvettes as the reference samples. By measuring both the excitation and emission spectra for both samples, the ratio between the numbers of photons emitted and absorbed by the experimental samples was derived, and thus the QY was obtained.

## Additional Information

**How to cite this article**: Jiang, Z. C. *et al.* A Facile and Low-Cost Method to Enhance the Internal Quantum Yield and External Light-Extraction Efficiency for Flexible Light-Emitting Carbon-Dot Films. *Sci. Rep.* **6**, 19991; doi: 10.1038/srep19991 (2016).
